# Food industry’s interactions with nutrition professionals in Australia: a survey

**DOI:** 10.1017/S1368980026103097

**Published:** 2026-07-09

**Authors:** Navid Teimouri, Katherine E. Sievert, Adam Hannah, Katherine Cullerton

**Affiliations:** 1 School of Public Health, The University of Queensland, Australia; 2 School of Exercise and Nutrition Sciences, Deakin University Faculty of Health, Australia; 3 School of Political Science and International Studies, The University of Queensland, Australia,

**Keywords:** Nutrition professionals, Food industry, Survey, Dietitian, Conflict of interest, Commercial determinants of health, Public health

## Abstract

**Objective:**

To identify interactions between the food, beverage and nutrition supplement industry and nutrition professionals (NP) in Australia.

**Design:**

The study employed a cross-sectional analysis of interactions by using an online survey including open and closed questions.

**Setting:**

Australia.

**Participants:**

156 practicing NP were recorded, 118 of them included in the analysis, 86 of which were dietitians.

**Results:**

Among respondents, eighty-eight (75 %) reported having previously received promotional material from industry, and seventy-four (66 %) have attended industry-sponsored educational events for professional development. Fifty-six (47 %) reported receiving gifts from industry in the past year. Of those who have received promotional or educational material, eleven (12 %) found it not useful at all, while others declared it slightly to extremely useful. Further analysis revealed that professional bodies and networks acted as intermediaries between industry and NP and that there were differences in judgement based on the specific industry they receive the material from.

**Conclusion:**

The Australian food, beverage and nutrition supplement industry is attempting to actively engage with NP to promote their products through trusted sources. NP’ opinions on this vary, and further research is needed to examine the scope and impact of these interactions.

The relationship between health professionals and commercial industries has long been a topic of debate and scrutiny, including beyond the scientific community. A growing body of evidence has demonstrated how various industry practices and engagement can shape professional practices, decision-making and public health policy in Australia^(1,2)^ and globally^(3–5)^. This also includes efforts by industry to produce and disseminate favourable research outputs^(6)^. However, most of this research has occurred in the context of the pharmaceutical industry and their engagement with physicians. More recently, attention has expanded to include the food industry, which similarly has strong commercial interests in shaping dietary behaviours and public perceptions of food and nutrition. In this context, foods and drinks harmful to health, such as ultraprocessed foods, and the political tactics used by this industry to promote them have become a major focus of public and academic debate^(7–10)^. Despite this increasing awareness and the importance of good nutrition for public health, limited empirical research exists on the extent and nature of industry interactions with nutrition professionals (hereafter NP)^(11)^, particularly in the Australian context. Existing research has largely focused on the food industry’s role in nutrition policy development, demonstrating influence on key policymaking decisions through access to decisionmakers^(12)^; however, there has been limited attention to its influence on nutrition professionals. Australia’s food system is highly industrialised with food manufacturing representing the nation’s largest manufacturing sector^(13,14)^. Nationally representative data indicate that ultraprocessed foods account for 42 % of total energy intake, contributing to a variety of detrimental public health outcomes^(15)^. Given the strong presence of food industry actors in Australia, many of the issues identified in physicians’ engagement with the pharmaceutical industry, such as biased prescribing behaviour or consultation, may also apply to other health professionals^(16)^ and their interaction with the food industry – yet this remains an important gap in the academic literature.

Dietitians, nutritionists and other health professionals who provide dietary advice play a central role in supporting public health agendas^(17,18)^. In Australia, dietitians are regulated professionals with protected titles requiring accredited university training and ongoing professional development, whereas nutritionists operate under an unregulated title with variable qualifications and no mandatory credentialing body^(19)^. The national dietetics workforce has expanded sevenfold over the past three decades, underscoring their growing importance as a credible source of nutrition information^(20)^. NP are also potential intermediaries between the food industry and the public, and their credibility can lend weight to nutritional or food recommendations. Hence, the food industry is likely to have a vested interest in cultivating and leveraging these relationships, to increase public trust in and ultimately consumption of their products^(21)^.

Hamel *et al.* ^(11)^ identify a range of active and passive interactions between NP and industry. Active interactions describe practices where both professionals and industry contribute to the relationship. This includes receiving compensation for endorsing industry products to clients or the broader public or being employed by industry directly or for consultation services^(11)^. Passive interactions do not involve direct engagement by NP, instead, they entail receiving targeted communication or gifts, samples and educational material from the industry^(11)^. The limited research on this issue indicates that NP have mixed opinions about interactions with the food industry. While they recognise potential benefits, such as career opportunities and the possibility of improving food quality, they are also aware of the risks to their professional independence^(9)^. Previous research has shown that important avenues of influence for industry are educational events facilitated through professional bodies^(11)^. In Australia, several professional bodies and networks represent or support NP (see Table 1). They differ in their scope, eligibility requirements for membership and the benefits they provide; this ranges from managing the accreditation needed to access government payments for services to providing professional development^(22)^. Hence, the relationships between industry, professional bodies and networks and NP are important to examine. Similar to attending industry-sponsored educational events, receiving industry gifts has been connected to influencing prescribing behaviour by health professionals in other fields, irrespective of the economic value of the gift^(23,24)^.

**Table 1.** Professional bodies for selection Table 1 long description.

Professional body	Description
Dietitians Australia (DA)	The peak professional body^*^ for dietitians in Australia, responsible for accrediting dietetic programs and maintaining the credential of Accredited Practising Dietitian (APD). Funded through membership fees, accreditation services and professional development programmes. It ceased partnerships with food industry from 2018 onwards.
Dietitian Connection (DC)	Privately owned professional platform that provides resources, events and communication channels for dietitians across Australia and international. Commercially funded through sponsorships, advertising and partnerships with industry.
Public Health Association of Australia (PHAA)	National peak body for public health professionals advocating for evidence-based policies on issues such as nutrition, disease prevention and health equity. Funded through membership fees, government grants, events and limited ethical sponsorship.
Sports Dietitians Australia	Leading professional body for sports dietitians. Funded by membership fees, course delivery and industry partnerships.
Nutrition Society of Australia	Scientific organisation promoting the advancement of nutrition science and research. Funded by membership fees, conferences and sponsorships from industry and academic partners.

* A peak body describes an umbrella organisation that represents and coordinates the interests of a particular profession or sector.

To date, however, little research has examined the nature and extent of these relationships. This study addresses a critical gap in the literature by providing the first empirical examination of interactions between NP and the food industry in Australia. Through a cross-sectional survey of dietitians, nutritionists and other relevant health professionals, the study explores their experiences with food industry communication, education, promotional materials and gift receiving. It also aims to capture attitudes and ethical considerations expressed by participants.

## Methods

We developed and distributed a self-administered, online survey to NP in Australia. The survey included quantitative and closed questions and matrices, as well as several open-ended questions for free-text responses. The survey was developed in Qualtrics^(25)^ in English language, and all participants needed access to a computer or a mobile device with suitable browsers to fill out the survey. Ethics approval was obtained through The University of Queensland Ethics Committee (approval number: 2024/HE000861).

### Survey development

The survey was designed to understand NP’ engagement with the food, beverage and nutrition supplement industry up to the past 10 years, to capture both recent and longer-term patterns of engagement. We used the term ‘food industry’ to include all commercial organisations across the food supply chain and related industry associations such as Dairy Australia. ‘Nutrition supplement industry’ was used to refer to companies that manufacture dietary supplements such as vitamins or minerals. The survey was developed in an iterative process involving multiple steps of testing and refining in the pilot phase. An initial set of questions was created based on a literature search and analysis of similar existing research^(26,27)^. The survey was pilot-tested by eleven dietitians, and feedback on question clarity, length, completion time and workforce structure, and regulation was incorporated into the revised survey. Demographic questions covered profession type, professional body or network membership and type of employment. A series of further questions evaluated the involvement of participants in professional development, media engagement and their experiences receiving and engaging with food, beverage or nutritional supplement industry-related communication. Participants were also asked about the food, beverage and nutritional supplement industries’ (hereafter referred to as ‘industry’) communication practices. The questions referred to the last 10 years as a timeframe and explored the frequency of communication by industry. The survey also included questions specific to the meat industry reflecting an early focus of the study. However, due to limited data, results are presented at the level of the food industry more broadly.

In this survey, five major professional bodies or networks related to nutrition were included for the participants to declare their memberships. An overview of each professional body or network can be found in Table 1.

The survey took approximately 10 minutes to complete in full. At the beginning of the survey, participants were provided with a description of the research project and a link to the participant information sheet and the consent information. Participant consent was implied through their commencement of the survey. The full survey can be found in online supplementary material, Supplemental File A.

### Participant recruitment

A multi-pronged approach was adopted with the aim of recruiting a diverse range of NP. While the focus was on NP, doctors also provide nutrition advice at times; hence, they were not excluded from the survey participants. First, the survey was distributed via professional networks of the research team, including promotional posts on social media networks LinkedIn and Bluesky. Second, several professional associations (Public Health Association of Australia, Australian Society of Lifestyle Medicine) agreed to distribute the survey invitation to their networks, mainly through newsletters. Third, because the distribution through networks and professional associations led to an overrepresentation of research dietitians, public online databases of practicing dietitians were used to contact dietitians across the country directly. Participants were eligible for inclusion in the study if they were health professionals who:

- provided dietary or nutritional advice to clients OR were trained as dietitians or nutritionists and;
- were based in Australia

All surveys were conducted anonymously. At the end of the survey, participants were given the option to receive a summary of the survey results. These e-mail addresses were not connected to their survey responses, and all responses remained anonymous. This was intended to support honest and open opinions by reducing concerns about identification or professional repercussions on this sensitive topic. The survey was open from 3 February until 29 April 29 2025.

### Data analysis

Survey responses were exported from Qualtrics into a Microsoft Excel spreadsheet. Responses where <65 % of the questions were completed, as well as those from participants not based in Australia, were excluded from analysis. Quantitative data were analysed using descriptive statistics, including frequencies and means. Cross-tabulation analyses were conducted using pivot tables to compare responses across groups. For free-text responses, data were exported to NVivo software, and an inductive content analysis was conducted. Responses reflecting similar ideas or issues were clustered together using *in vivo* coding where possible, enabling the identification of common themes. Relevant illustrative quotes were identified as part of this analysis.

## Results

A total of 156 participant responses were recorded by Qualtrics. After removing incomplete surveys and participants not based in Australia, 118 responses were included for data analysis. Most participants were dietitians (73 %), followed by nutritionists (22 %) and researchers (19 %). Table 2 provides an overview of demographics regarding professional information.

**Table 2.** Demographics of participants Table 2 long description.

Category	*n* ^*^	%
Profession	118	100
Dietitian	86	73
Nutritionist	26	22
Researcher	22	19
Doctor	3	3
Other^†^	11	9
Dietitian domain	86	100
Dietitian in private practice	46	53
Public health dietitian	18	21
Research dietitian	16	19
Community dietitian	14	16
Dietitians in hospital	9	10
Dietitians working with food industry	3	3
Other	12	14
Type of employment	118	100
Self-employed	49	42
Academia	37	31
Government organisation	28	24
NGO	16	14
Commercial organisation	15	13
Other	8	7
Membership in professional body	118	100
Dietitians Australia	84	71
Dietitian Connection	55	47
Public Health Association of Australia	29	25
Nutrition Society of Australia	19	16
Sports Dietitians Australia	17	14
Other	22	19
Years of professional experience	118	100
0–2 years	21	18
3–5 years	25	21
6–10 years	23	19
Over 10 years	49	42

* Numbers may add up to above 100 % because participants were given the option to choose multiple answers.

† Five of these also chose dietitian as profession, one worked for government, two were dentists and three worked for non-government organisations in unclear professions.

### Industry communication

Participants were asked about the communication strategies used by industry to engage with them. Seventy-one percent of respondents declared that they had previously received direct communication from industry (i.e. emailed to them directly), and for 52 % of respondents, this type of engagement is ongoing. Moreover, 43 % reported having been asked by industry to promote specific products. Table 3 provides a more detailed overview of the responses.

**Table 3.** Communication by F&B and nutritional supplement industry Table 3 long description.

*n* 118 (%)	Have received		Still receiving		Never received		Not sure	
	*n*	%	*n*	%	*n*	%	*n*	%
Direct communication from F&B or nutritional supplement industry	84	71	61	52	26	22	8	7
Promotional material from association that promoted F&B or supplements	88	75	64	54	13	11	17	14
Approached by F&B or nutritional supplement industry	70	59	55	47	40	34	8	7
Asked to promote products by F&B or nutritional supplement industry	51	43	32	27	61	52	6	5

For all four questions regarding the frequency of industry communication, direct contact typically ranged from monthly to 1–2 times per year. Only a small number of respondents (2–10 %) reported receiving this communication weekly or more frequently. When comparing responses by type of employment, self-employed respondents reported receiving communication more frequently compared with the overall group (53 % *v*. 45 % reporting communication yearly or more frequent across all four questions), while those working in academia reported receiving less (35 % *v*. 45 %).

Several respondents commented that Dietitians Australia had previously engaged in industry partnerships but has since stopped this practice. Some also noted that Dietitian Connection provided a significant amount of industry-sponsored material. However, several participants indicated that they were not receiving industry-related communication as they actively unsubscribe from such content. For example, one respondent noted that ‘*The reason I don’t get much industry sponsored content is more related to the fact that I actively unsubscribe from that kind of content or don’t open those types of emails etc*.’

For those receiving industry-sponsored material, frustration was voiced by some with professional networks promoting industry-sponsored content to NP. One respondent stated that they subscribed to a specific professional network ‘*only to see the extent of industry manipulation of dietetic practice. That service seems to be wholly bought and paid for by industry and I am appalled by the rubbish they promote.*’

### Professional education

Of the 118 included respondents, 112 reported participating in regular professional development through courses, workshops, seminars or similar. Of these, seventy-four (66 %) had attended at least one food industry-sponsored educational event in the last 10 years. There was a large variety of industries, companies and associations sponsoring such professional development, but frequently mentioned food corporations included large food processing and nutrition supplement firms such as Nestle (*n* 11), Abbott (*n* 7) and Nutricia (*n* 8) as well as broader industry representative groups such as Meat and Livestock Australia (*n* 14) and Dairy Australia (*n* 7). Of note, while most respondents reported attending industry-funded professional development previously, only 17 (14 %) reported using industry information sources to inform their dietary guidance. Other sources of information used were scientific journals (90, 76 %), trainings/seminars and similar (78, 66 %), professional organisations (72, 61 %), social media (26, 22 %) and traditional media (13, 11 %).

The most frequently mentioned professional organisations that were used for professional education were Dietitians Australia, Dietitian Connection, Public Health Association of Australia and different national and international conferences. Respondents who reported using social media for information mostly used LinkedIn, X and Instagram, predominantly following other NP as well as nutrition influencers. Additional information sources reported by participants were colleagues, supervisors or internal professional development. For those using industry-specific resources to inform their work, newsletters from specific companies or industry associations were commonly mentioned, such as the Nuts for Life newsletter. There were no differences in sources of education used across the different professions or types of dietitians.

### Gifts and event invitations

Approximately half of all participants (*n* 56, 47 %) reported receiving gifts or invitations from industry in the past year, including from industry associations such as Meat and Livestock Australia. Food and beverage product samples and merchandise were the most popular gifts (see Table 4).

**Table 4.** Gifts received from industry Table 4 long description.

Type of gift received	*n* 117	
	*n*	%
Food/beverage product as a sample	47	40
Merchandise, e.g. stationary, mouse pads or similar	20	17
Food/beverage at the workplace, e.g. provision of lunch	8	7
Honoraria for speaking	7	6
Payment for consulting services	6	5
Costs of travel, time, meals, registration or similar for attending conferences	3	3
Tickets to events including private events	2	2
Other^*^	8	7

* For example sponsored sessions including food and beverages at conferences or invitations to present at industry events.

### Use of promotional/educational industry material

Seventy-eight percent of participants (*n* 90) reported receiving some form of promotional or educational material from industry, such as patient handouts/leaflets or information sheets. Of these, 11 (12 %) said they did not find the material useful at all, while forty (44 %) found it slightly useful, twenty-nine (32 %) moderately useful and ten (11 %) very or extremely useful. Content analysis of how participants use this material revealed several different purposes.

The most stated use of industry material was for their own general education (*n* 27). This included staying up to date with recent research, understanding industry developments regarding their products or gaining general nutrition knowledge. Some respondents also described using the materials to monitor industry strategies to promote their products. For example, one respondent commented that they use the resources to: ‘*… scan for current research, and understand which research…industry is taking note of. The resources can be helpful in understand how different [stakeholders] (e.g. food industry) [are] framing particular issues*.’ A number of respondents recognised the potential bias industry materials may contain.

Twenty-three respondents reported providing industry-sponsored material to their clients. Resources such as meal planners or leaflets describing the nutritional information of individual products were singled out as particularly useful. Respondents also reported providing their clients with product samples, particularly nutritional supplements, to help them assess their effectiveness. Two respondents declared that they distribute such materials only after informing the clients that the information is from industry sources and should be treated with caution. For example, one respondent wrote that they do: ‘*give it [industry sponsored material] to clients, but after explaining that it is MLA/Dairy Australia/etc promotional material and therefore to take due consideration that there could be vested interests and not the whole truth*’. Nonetheless, this perspective was stated infrequently, so it is unclear whether respondents are providing additional explanation or framing of the material prior to sharing it with clients.

One common use of industry resources was to obtain updated product information (*n* 17), for example, nutritional values of individual products, details of new products released or to learn about the taste of the products. The material was also used as inspiration by some (*n* 10), including for preparation/use of meal planners, recipes and informational materials were used to inform future research and to create their own resources. A further seven respondents reported using the material for personal use, including food products and merchandise, such as USB flash drives. Lastly, twenty participants expressed scepticism of any industry material they receive, citing concerns about potential bias in the material and the use of excessive marketing for some products. For example, one argued that such materials *‘are by definition not objective and thereby fatally compromised in terms of trustworthiness. I would never trust such material.’* Others also cited ethical concerns when explaining why they don’t use the material, with one respondent stating: ‘*Resources such as meal planners are great tools to use. If they didn’t have the marketing elements, I would use them. I don’t use them because I feel it is against my ethos but I do get good ideas for resource development*.’

### Organisational policies

Of the 114 respondents to this question, 43 (38 %) reported that their organisation/association had policies or guidelines about interacting with industry, for example, universities or research institutes. Forty-four (39 %) were unsure whether such polices existed, and another 27 (23 %) said there were no policies or guidelines in place. Many of the respondents reporting the existence of organisational policies were research dietitians whose universities had established policies on interacting with industry. While some policies did not allow accepting gifts from industry, most followed a transparency approach where gifts over a certain value need to be declared and sometimes made publicly available. Some organisations had informal restrictions on receiving industry sponsorship, as one pointed out: ‘*Unwritten policy- We do not accept any promotions from companies that we do not actively use or recommend for our clients from an evidenced based and unbiased perspective. As of yet, we have not taken any sponsorships or funding as there are very few companies that fit into this category.*’ Others appeared to make personal decisions about accepting gifts: ‘*I do not accept gifts or promotions from industry other than samples for me to review in order to understand their benefits*.’

### Complexity and transparency in industry-professional relations

In the open-text responses, several respondents reflected on the diversity of the food industry and the complexity of industry–NP relationships. One highlighted the distinction between ‘Big Food’ and other food companies: ‘*I think it’s really important to consider diversity within ‘food industry’. I have taken these questions in the context of ‘big food’.*’ Similarly, multiple comments throughout the survey emphasised the need to differentiate between the primary producers and food manufacturers, such as this one: ‘*I have no qualms about sharing materials from Australia’s primary produce industry – Australians are not eating enough of these foods. I do however think you should be asking about the manufacturing sector and nutraceutical and pharmaceutical companies*.’

Further, some pointed out that food industry material may be disseminated through other organisations such as public relations companies or even government agencies such as the Australian Commonwealth Scientific and Industrial Research Organisation. There were concerns that the use of professional bodies and intermediary organisations by the food industry can obscure the visibility of industry material and hinder health professionals from clearly identifying it. This complexity is described by one respondent: ‘*Perhaps [consider] unpacking people’s understanding of food industry visibility in these activities; sometimes they aren’t clearly visible/stated (e.g. industry front groups) or scenarios where the person may have received information or attended a webinar and didn’t realise it was industry-funded. This is particularly pertinent for emerging health professionals who may not have an awareness of the widespread nature of industry lobbying and involvement in shaping the conversations/debates/controversies in food industry*.’

Overall, opinions on interacting with industry were mixed. Some respondents preferred industry to be completely absent: ‘*Dietetic professionals who are paid by industry should not be given platforms at conferences. By definition their objectivity is compromised so any claims they make are not to be trusted*.’ Others pointed out that industry and health professionals may have shared goals to work towards: ‘*It’s really tricky to navigate. Some of the situations are black and white, but some are not. I tend to avoid as much as possible for the sake of my career, but part of me also thinks if all sectors stopped demonising each other it wouldn’t necessarily be a bad thing for any shared goals.*’ To improve trust in industry material and collaboration between health professionals and the food industry, some respondents also called for policy: ‘*Tighter regulation and transparency with the food and beverage industry might build more community trust. I would like to engage more with industry but I am also wary of reputational damage*.’

## Discussion

Our findings highlight considerable engagement between NP and the food industry in Australia, which may influence professional practice and decision-making. Two-thirds of participants reported attending a food industry sponsored educational event in the last 10 years for professional development. About half of respondents currently receive direct communication from the food industry, and over 70 % declared that they have received it previously. A similar percentage of respondents had received promotional material, and over half had been directly asked to promote food industry products. Many of the respondents also indicated regularly receiving gifts from the food industry. This included foods and beverages, stationery or financial compensation for attending events. These findings illustrate evident engagement between NP and industry, although, experiences, perceptions and opinions on this engagement vary widely.

Most participants in the study took part in industry events for their professional development. This aligns with the results of two recent research projects by Hamel *et al.* ^(9,11)^. Their global literature review and subsequent survey among Canadian NP identified industry involvement in professional education as a key avenue of interaction^(9,11)^. Influencing the education of health professionals for commercial benefit is a well-researched strategy used by different industries^(6)^. In Australia, continuing professional education is an important component of development for NP. To maintain accreditation, an Accredited Practising Dietitian needs to complete 30 h of professional development each year, typically by taking relevant professional development courses/workshops^(28)^. These can be provided by industry actors. Research from other countries highlights that food industry-sponsored professional development opportunities often blend educational content with subtle promotional messaging^(21,29,30)^. Similar strategies have been observed in other industries, particularly the pharmaceutical industry^(1)^, where evidence indicates that even small-scale engagement with industry through educational events is likely to affect professional recommendations^(4,31,32)^. These effects are often subconscious and influence choices around recommending products to clients in the future^(23,33)^.

The involvement of professional bodies and networks in providing education events highlights their role as influential intermediaries that can legitimise and normalise industry participation in NP education. Previous research has similarly identified professional organisations as important facilitators of industry communication and professional development events targeting NP^(11)^. The contrasting approaches taken by professional platforms in Australia illustrate how organisational policies can either constrain or enable such influence. While Dietitians Australia ceased collaborating with the food industry following ongoing criticism^(34)^, other professional networks continue to offer a range of professional development courses, information materials and patient resources, many of which are supported by industry actors such as Nestle, Abbott, Arnotts, Eggs Australia, Meat and Livestock Australia and Nuts for Life^(35–37)^. Notably, these organisations’ websites actively promote advertising opportunities for food brands and corporate partners, emphasising the opportunity to reach 99 % of dietitians in Australia and the potential for industry products to be promoted by them to their clients^(38)^. Partnerships between professional bodies and the food industry have previously been observed in many countries^(11)^. The American Academy of Nutrition and Dietetics, the world’s largest organisation of food and nutrition professionals^(39)^, has been criticised for its close alignment with industry positions in some policy venues and financial ties to food industry actors such as Nestle and PepsiCo^(40)^. The American public health transparency organisation, U.S. Right to Know, highlights the dangers of such industry ties as they threaten and counteract the public health agenda of organisations like the Academy of Nutrition and Dietetics are meant to support and may undermine public trust of by providing academic and professional credibility to potentially harmful products^(39,40)^.

For the food industry, professional development events serve as opportunities to maximise profits and shareholder value, which is often prioritised at the expense of beneficial public health outcomes^(21)^. Published research and development reports from MLA bring further clarity as to the benefits of these partnerships for industry. For example, a recent MLA report on a partnership with Dietitian Connection states that it undertook a *‘promotional campaign with dietitians to ensure consumers are confident to enjoy red meat in three to four balanced meals a week through consistent messaging from trusted sources’* ^(41)^. NP are considered trusted sources that are important facilitators of industry’s consumer messaging. Our survey results show that different industry actors, ranging from global food manufacturers like Nestle to national industry associations, have offered educational events in co-operation with certain professional bodies or networks. While our results highlight that a large proportion of NP attend industry events, they do not provide information on the exact number of events or total participants. Whereas an analysis of pharmaceutical industry-sponsored events in Australia revealed that over 600 events were held per week, each with 30 attendees on average, with total attendances exceeding 3 million across the 4-year study period^(1)^. A similar analysis for the food industry may be beneficial in identifying the scope and reach the food industry has among NP. This would complement our analysis to not only understand which type of engagement occurs but to also provide data on the national scale of engagement. Further research could also explore how widespread financial ties between the food industry and NP bodies are and what impact they have, as seen in research in professional medical associations^(42)^.

Given that over half of NP in our survey reported receiving gifts from industry in the past year, this could also be an avenue of influence for industry to promote their products and increase their sales^(23,24)^. For example, one study found that gifts from pharmaceutical firms to physicians result in them prescribing more branded medications, leading to higher prescription costs^(24)^. Further, gift receiving has been associated with inappropriate prescribing of drugs, and there have been calls for all industry gifts to be banned^(24,43)^. This practice can influence patient perceptions of their physicians. Patients who believe gift receiving is common among physicians report lower levels of physician trust and generally higher levels of health system distrust^(44)^. Similar dynamics could result in decreased trust in nutrition care and the public health agenda. Future research could investigate whether these mechanisms are applicable to relationships between the food industry and NP.

Our results regarding the use of industry-produced promotional materials provide some insight into the impact they may have on NP. Many respondents reported using the material for their own education and knowledge or to stay up to date with current research. Given that industry-produced materials may lack a comprehensive and balanced overview of nutritional benefits and risks, their use could influence the knowledge base for NP^(11)^. This is further compounded by industry creating and disseminating targeted and potentially biased research to promote their products^(6)^. Notably, a substantial number of NP passed this material on to their clients. This highlights a need for accessible, high-quality resources NP can use for their knowledge and to pass on to clients. Increasing the availability of independent, evidence-based resources may lower the dependency on industry resources. As previously discussed, there are risks associated with the use of industry resources, including a potential disparity between industry’s and patients’ interests^(45)^, diminished trust in the health system^(46)^ and increased exposure of NP to industry-preferred foods^(47)^. Another risk of collaboration with industry is that it may discourage NP or their associations from voicing criticism towards industry or limit critical thinking regarding industry practices^(48)^. These risks are part of the broader discussion around commercial determinants of health, which describe the adverse planetary and human health impacts products and practices of some commercial actors are producing^(49)^.

Nonetheless, there were calls from respondents to acknowledge the complexity of the food industry and to differentiate between different food industry actors, with several expressing particular criticism of food manufacturers and multinational corporations. Similar findings have been found in the USA and in previous research in Australia, where NP were generally against partnerships with large corporations and food manufacturers, particularly those producing processed food, but were less critical about primary produce associations^(27,50)^. This distinction in ‘good’ and ‘bad’ industry actors is noteworthy, particularly in the context of environmental sustainability and social and animal ethics. While products supplied by primary producers directly may be healthier than processed foods, large-scale farming, and particularly animal agriculture, is often associated with severe environmental impacts such as greenhouse gas emissions, deforestation and excessive land and water use^(51)^, as well as social harms such as poor labour practices and excessive market power in food supply chains^(52,53)^. While NP’ primary focus is on human dietary health, which can make them less attuned to the broader environmental, social and ethical harms associated with primary production, this does not mean that engagement with these actors would be without risk.

Further, some respondents identified perceived benefits of industry interaction for NP. This aligns with previous literature, where financial benefits, direct access to products, information and educational events or the industry’s reach to the public were seen as potential benefits^(11)^. Only a small proportion of respondents expressed frustration with industry produced material and communications. Previous research indicates that while some practitioners are sceptical about partnerships and communication with industry, attitudes vary widely, with many practitioners or professional associations accepting or even encouraging industry engagement^(11,26)^. Given the well-documented risks of industry collaboration, these findings suggest that increased efforts in education and awareness building regarding the dangers of industry involvement with health professionals may be necessary.

One phenomenon that was not surveyed but was mentioned by several respondents is the rise of dietitian influencers on social media and the need for close attention to their sponsors. Investigative journalism from the Washington Post recently reported that the food industry paid dietitian influencers to encourage ultraprocessed foods high in sugar or artificial sweeteners^(54)^. These posts counter decades of scientific evidence and reach millions of followers on social media^(54)^. This may necessitate more attention or guidelines among the scientific research community globally, but also in Australia specifically^(55)^.

Given the risks associated with interactions between the food industry and NP, pre-emptive measures to mitigate them may be warranted. Guidelines or codes of ethics/engagement issued by authoritative bodies like professional associations or public institutions could provide clarity for NP if they include specific decision-making criteria for managing different types of interactions, particularly as some decisions are made subconsciously^(9,55)^. This is particularly relevant for nutritionists who are unregulated compared with dietitians and doctors and may require clear guidance on industry engagement. A toolkit similar to the FoRK guidance and toolkit by Cullerton *et al.* ^(56)^, which provides guidance to food and nutrition researchers working with industry, could be adapted for NP. Further, the decision from Dietitians Australia to cease collaborations with the food industry significantly reduced industry communication with NP through their network^(34)^. While this was an important step forward, other networks such as Dietitian Connection have filled this gap. Hence, stricter regulation and explicit guidelines may be more effective than voluntary disengagement. More broadly, reducing food industry influence on nutrition policy more generally remains an important lever for mitigating the harmful effects of industry involvement in nutrition and public health in Australia^(57)^.

This study has several strengths. To our knowledge, this is the first study to survey Australian NP about their relationships with the food industry. It surveyed a variety of interaction possibilities between industry and NP and allowed for a mixture of quantitative and open-text responses. Further, professionals from diverse demographic backgrounds, occupations and association memberships participated, enabling data collection across a broad range of NP with different geographical background and employment situations. There are also limitations. The sample was small in comparison to the total number of NP in Australia. It also included very few general practitioners or specialist doctors, who may also provide nutritional advice. Targeting these practitioners could be a focus for future research. Additionally, there were fewer respondents from the Nutrition Society of Australia, which has a more open stance on food industry sponsorship. Higher response numbers from government-employed, industry-employed and hospital-based NP may have added valuable depth, and future studies might consider contacting employers directly to reach these groups. Finally, social desirability and recall bias may have influenced responses as participants may have had a particular interest in these issues. Relatedly, NP with stronger industry alignments may have been less likely to participate.

### Conclusion

While there is a large body of research that has investigated the effects of the pharmaceutical industry’s attempts to engage with health professionals, the food industry has remained largely unscrutinised. This study provides the first empirical examination of the nature and extent of interactions between NP and the food industry in Australia. The findings demonstrate substantial engagement across multiple channels, including educational events, direct communication and through professional bodies. While some NP perceive benefits from such engagement, the potential for these interactions to influence their practice, shape dietary advice and undermine public trust raises important public health concerns. Given the parallels with strategies employed by other industries, particularly pharmaceutical, these findings underscore the need for more scrutiny and research. Future research could quantify the scale of industry engagement and its impacts on professional practice, drawing on examples from research on pharmaceutical industry practices.

## Supporting information

10.1017/S1368980026103097.sm001 Teimouri et al. supplementary material Teimouri et al. supplementary material

## Supplementary material

For supplementary material accompanying this paper visit https://doi.org/10.1017/S1368980026103097

## Table 1. Long description

A table listing professional bodies for dietitians in Australia and their roles. The table has five rows and two columns. The first column lists the names of the professional bodies, and the second column describes their roles. Row 1: Dietitians Australia (DA), The peak professional body for dietitians in Australia, responsible for accrediting dietetic programs and maintaining the credential of Accredited Practising Dietitian (APD). Funded through membership fees, accreditation services and professional development programmes. It ceased partnerships with food industry from 2018 onwards. Row 2: Dietitian Connection (DC), Privately owned professional platform that provides resources, events and communication channels for dietitians across Australia and international. Commercially funded through sponsorships, advertising and partnerships with industry. Row 3: Public Health Association of Australia (PHAA), National peak body for public health professionals advocating for evidence-based policies on issues such as nutrition, disease prevention and health equity. Funded through membership fees, government grants, events and limited ethical sponsorship. Row 4: Sports Dietitians Australia, Leading professional body for sports dietitians. Funded by membership fees, course delivery and industry partnerships. Row 5: Nutrition Society of Australia, Scientific organisation promoting the advancement of nutrition science and research. Funded by membership fees, conferences and sponsorships from industry and academic partners.

Navigate back to Table 1..

## Table 2. Long description

The table presents participant demographics across various categories. It has 11 rows and 3 columns. The columns are labeled Category, n*, and %. The table includes data on profession, dietitian domain, type of employment, membership in professional body, and years of professional experience. Each category is broken down into subcategories with corresponding values for n* and %. For example, under the Profession category, there are subcategories like Dietitian, Nutritionist, Researcher, Doctor, and Other, with values for n* and % provided for each. The table provides a detailed breakdown of the participants' professional information.

Navigate back to Table 2..

## Table 3. Long description

A table with four rows and five columns detailing communication strategies used by the F&B and nutritional supplement industry. The columns are labeled as Have received, Still receiving, Never received, and Not sure, with sub-columns for count (n) and percentage (%). The row labels are Direct communication from F&B or nutritional supplement industry, Promotional material from association that promoted F&B or supplements, Approached by F&B or nutritional supplement industry, and Asked to promote products by F&B or nutritional supplement industry. Row 1: Direct communication from F&B or nutritional supplement industry. Have received: n 84, 71 percent; Still receiving: n 61, 52 percent; Never received: n 26, 22 percent; Not sure: n 8, 7 percent. Row 2: Promotional material from association that promoted F&B or supplements. Have received: n 88, 75 percent; Still receiving: n 64, 54 percent; Never received: n 13, 11 percent; Not sure: n 17, 14 percent. Row 3: Approached by F&B or nutritional supplement industry. Have received: n 70, 59 percent; Still receiving: n 55, 47 percent; Never received: n 40, 34 percent; Not sure: n 7, 6 percent. Row 4: Asked to promote products by F&B or nutritional supplement industry. Have received: n 51, 43 percent; Still receiving: n 32, 27 percent; Never received: n 61, 52 percent; Not sure: n 6, 5 percent.

Navigate back to Table 3..

## Table 4. Long description

A table titled 'Type of gift received' with data on the frequency of different types of gifts received. The table has 117 entries and includes the following columns: Type of gift received, n, and percentage (%). The rows are as follows: Row 1: Food/beverage product as a sample, 47, 40 percent. Row 2: Merchandise, e.g., stationary, mouse pads or similar, 20, 17 percent. Row 3: Food/beverage at the workplace, e.g., provision of lunch, 8, 7 percent. Row 4: Honoraria for speaking, 7, 6 percent. Row 5: Payment for consulting services, 6, 5 percent. Row 6: Costs of travel, time, meals, registration or similar for attending conferences, 3, 3 percent. Row 7: Tickets to events including private events, 2, 2 percent. Row 8: Other, 8, 7 percent.

Navigate back to Table 4..
