# Supplementary material for: Food industry’s interactions with nutrition professionals in Australia: a survey
Source: Public Health Nutr. 2026 Jul 9;29(1):e158. doi: 10.1017/S1368980026103097 (PMC13434609; doi:10.1017/S1368980026103097)
Supplement: Supplementary file 1 [file S1368980026103097sup001.docx]

Trial study 2

Start of Block: Introduction

Intro Thank you for your interest in participating in this research project. Please read the following information about the project so you can decide whether you would like to take part.<br> <br> This survey aims to understand the relationships of health professionals with industry partners from the food and beverage industry (including nutrition supplement companies). By completing this survey, you consent to having your data used for research purposes. The findings of this project may be published in peer-reviewed research journals. All data provided by you is <strong>anonymous</strong>. If you wish to review the participant information sheet and the consent information, you can click <a href="https://cloud.rdm.uq.edu.au/index.php/s/y9fzwzdSCCT4qMk" target="_blank" rel="noopener noreferrer">here</a> (password: 'surveyindustry'). The survey should take 10-15 minutes.<br> <br> Participation in this research is voluntary. If you do not wish to take part, you do not have to. If you decide to take part and later change your mind, you are free to stop at any time. If you choose to stop participating, your data will not be used in this research. You will not receive reimbursement for the time taken to complete this survey.<br> <br> This survey has been prepared as part of a research project in the School of Public Health, University of Queensland, Australia. Ethics approval has been granted by The University of Queensland Human Research and Ethics Committee (Reference number: 2024/HE000861). If you proceed with completing this survey, your consent is implied.

End of Block: Introduction

Start of Block: Demographics

Demographics First, we will ask you some questions about yourself and your professional background.

Country of residence Which country are you based in?

- Australia
- Other __________________________________________________

Profession What is your profession?

- Dietitian
- Nutritionist
- General practitioner
- Specialist doctor
- Researcher/educator
- Other health professional
- Other __________________________________________________

Display this question:

If What is your profession? = Dietitian

Dietitian specialty What area of practice would you classify yourself as? (tick as many as relevant)

- Dietitian working in a hospital
- Dietitian working with/in food industry (this includes food & beverage companies including nutrition supplement companies)
- Research dietitian
- Community dietitian
- Public health dietitian/nutritionist
- Dietitian working in private practice
- Other __________________________________________________

| Page Break |  |
| --- | --- |

Years in profession How long have you been working in this profession?

- 0-2 years
- 3-5 years
- 6-10 years
- 10+ years

Type of organisation Where are you employed? If applicable, tick multiple boxes.

- Self-employed (e.g. private practice)
- Employed by a commercial/private sector company (e.g. Food and Beverage industry, private hospital)
- Government organisation (incl. public hospitals)
- Academia (e.g. university)
- Non-government organisation (e.g. Charity)
- Other __________________________________________________

Professional organis Are you part of any professional associations or network/s? If applicable, tick multiple boxes.

- Dietitians Australia
- Sports Dietitians Australia
- Nutrition Society of Australia
- Public Health Association Australia
- Dietitian Connection
- Other __________________________________________________
- None

End of Block: Demographics

Start of Block: Med education

Prof education The following section collects information regarding your ongoing professional education and information sources.

Med education Do you engage in ongoing continuing professional education in the area of dietetics/nutrition through courses, workshops, conferences, or similar?

- Yes
- No

Display this question:

If Do you engage in ongoing continuing professional education in the area of dietetics/nutrition thr... = Yes

Med edu yes In the last ten years, do you recall attending any professional education programs/workshops/seminars that were sponsored by the food industry (or nutrition supplement industry)? If yes, could you please list any of the sponsors/hosts of these events that you remember in the text box?

- Yes __________________________________________________
- No
- Not sure

| Page Break |  |
| --- | --- |

Science Do you believe you have enough knowledge about the latest nutrition research to effectively inform your work?

- Yes
- No

Display this question:

If Do you believe you have enough knowledge about the latest nutrition research to effectively infor... = Yes

Science yes Where do you get relevant nutrition and dietetic information to inform your practice? <br>Providing the name of the respective association/media etc. will be sufficient. <br>

- Scientific journals
- Professional organisations e.g. Public Health Association Australia (please specify) __________________________________________________
- Media e.g. television, radio, print or online media (please specify) __________________________________________________
- Food industry e.g. representatives, statements, newsletters (please specify) __________________________________________________
- Social-media e.g. X (Twitter), Facebook, Instagram (please specify) __________________________________________________
- Conferences, trainings, or events (please specify - in this case the title and provider/sponsor would be beneficial) __________________________________________________
- Other __________________________________________________
- Not sure

End of Block: Med education

Start of Block: Industry connections

Matrix F&B We will now look at food and beverage (F&B) industry practices to communicate with health professionals. F&B industry includes any food and beverage companies, including nutrition supplement companies or professional associations, such as Dairy Australia.<br>The following matrix explores communication practices between the F&B industry and health professionals. For this question, communication refers to unsolicited communication like promotional material, conference invitations, or anything similar. Please refer to the last ten years as a timeframe.

|  | Never | In the past, but not anymore | 1-2 times/year | 3-6 times/year | monthly | weekly | >1 per week | Not sure |
| --- | --- | --- | --- | --- | --- | --- | --- | --- |
| Have you directly received communication from the F&B industry? E.g. newsletters, briefings etc. |  |  |  |  |  |  |  |  |
| Have you received promotional material from a professional association (e.g. Dietitians Australia) that promoted specific foods, beverages or nutrition supplements. |  |  |  |  |  |  |  |  |
| Have you (or your organisation) been approached by any F&B industry (includes trade associations, companies, etc.) representatives in your professional role for any reason? |  |  |  |  |  |  |  |  |
| Have you (or your organisation) been asked by the F&B industry to promote their products? |  |  |  |  |  |  |  |  |

| Page Break |  |
| --- | --- |

Matrix meat <em>We now want to take a look at whether you have had any communication with the meat industry. </em>The following matrix includes the same questions as the one before specific to the<strong> meat industry</strong>. Meat industry in this case includes corporations (e.g. JBS), their brands (e.g. Primo), professional associations (e.g. Meat and Livestock Australia), farming associations (e.g. National Farmers Federation), or any other groups related to the meat industry. Please refer to the last ten years as a timeframe.<br /> If you have chosen never on all items of the previous matrix, your choice here should be never on all items as well.

|  | Never | In the past, but not anymore | 1-2 times/year | 3-6 times/year | monthly | weekly | >1 per week | Not sure |
| --- | --- | --- | --- | --- | --- | --- | --- | --- |
| Have you directly received communication from the meat industry? E.g. newsletters, briefings etc. |  |  |  |  |  |  |  |  |
| Have you received promotional material from a professional association (e.g. Dietitians Australia) that promoted meat or meat based supplements? |  |  |  |  |  |  |  |  |
| Have you (or your organisation) been approached by any meat industry representatives in your professional role for any reason? |  |  |  |  |  |  |  |  |
| Have you (or your organisation) been asked by the meat industry to promote their products? |  |  |  |  |  |  |  |  |

Further comm Matrix Do you have any further comments regarding the two matrixes?

________________________________________________________________

| Page Break |  |
| --- | --- |

Display this question:

If We now want to take a look at whether you have had any communication with the meat industry. The... != Have you directly received communication from the meat industry? E.g. newsletters, briefings etc. [ Never ]

Or We now want to take a look at whether you have had any communication with the meat industry. The... != Have you received promotional material from a professional association (e.g. Dietitians Australia) that promoted meat or meat based supplements? [ Never ]

Or We now want to take a look at whether you have had any communication with the meat industry. The... != Have you (or your organisation) been approached by any meat industry representatives in your professional role for any reason? [ Never ]

Or We now want to take a look at whether you have had any communication with the meat industry. The... != Have you (or your organisation) been asked by the meat industry to promote their products? [ Never ]

Contact meat industr In the last ten years, have you had any professional relationship with the meat industry? For this question, professional relationship refers to online or phone contact, meetings, conference attendance, media engagement, or anything similar. <br>

- Yes
- No

Display this question:

If In the last ten years, have you had any professional relationship with the meat industry? For thi... = Yes

Type of contact text Could you please elaborate on the type of contact. This may include meetings/phone calls with representatives, conference or workshop attendance, online communication, media engagement, or similar.

________________________________________________________________

| Page Break |  |
| --- | --- |

Gift receiving F&B We would now like to ask a question about engagement with the F&B industry generally.<br>In the last year, which of the following have you received from the F&B industry, including nutrition supplement companies or professional associations, such as Dairy Australia.

- Food/beverage in your workplace e.g. provision of lunch
- Food/beverage as samples
- Honoraria for speaking
- Payment for consulting services
- Costs of travel, meals, time, accomodation, registration or other for attending meetings/conferences
- Merchandise e.g. stationary, mouse pads or similar
- Tickets to events including private events such as concerts
- None
- Other __________________________________________________

Display this question:

If We would now like to ask a question about engagement with the F&B industry generally. In the last... != None

Gift receiving meat In the previous question, your response indicated that you have received products or payment from the F&B industry. We would now like you specifically about the <strong>meat industry</strong>.<br /> In the last year, which of the following have you received from a meat related company or association, such as Meat and Livestock Australia.

- Food/beverage in your workplace
- Food/beverage as samples
- Honoraria for speaking
- Payment for consulting services
- Costs of travel, meals, time, accomodation, registration or other for attending meetings/conferences
- Merchandise e.g. stationary, mouse pads or similar
- Tickets to events including private events such as concerts
- None
- Other __________________________________________________

| Page Break |  |
| --- | --- |

Material useful If you have ever received or been given <strong>promotional or educational</strong> material from the food or beverage industry, including the meat industry and nutrition supplement industry, how useful do you find this material?

- Not at all useful
- Slightly useful
- Moderately useful
- Very useful
- Extremely useful
- None received

Display this question:

If If you have ever received or been given promotional or educational material from the food or beve... != None received

Material engagement If you have ever received or been given <strong>promotional or educational</strong> material from the food or beverage industry, what do you use it for?

________________________________________________________________

________________________________________________________________

________________________________________________________________

________________________________________________________________

________________________________________________________________

| Page Break |  |
| --- | --- |

Policies Do you or your organisation/association have any policies/guidelines about interacting with industry or accepting gifts from industry?

- Yes
- No
- Not sure

Display this question:

If Do you or your organisation/association have any policies/guidelines about interacting with indus... = Yes

Policies yes Can you please outline below the policy and who has established this? If available, you may add a hyperlink to the text box. Any hyperlinks provided will be de-identified.

________________________________________________________________

| Page Break |  |
| --- | --- |

Comments Do you have any additional comments about this topic or survey? Are there any questions we should be asking but have not?

________________________________________________________________

| Page Break |  |
| --- | --- |

End of Block: Industry connections

Start of Block: Follow-up interviews

Following this survey, we will hold interviews with participants who have engaged with the food industry, including the meat industry. Do you have experience with industry engagement and would like to participate in one of these interviews?

- Yes
- No

End of Block: Follow-up interviews

Start of Block: Result distribution

Results Do you want to receive a short summary of the results after we have analysed the data from this survey?

- Yes
- No

End of Block: Result distribution
